# On the Cutting Performance of Segmented Diamond Blades when Dry-Cutting Concrete

**DOI:** 10.3390/ma11020264

**Published:** 2018-02-09

**Authors:** A. J. Sánchez Egea, V. Martynenko, D. Martínez Krahmer, L. N. López de Lacalle, A. Benítez, G. Genovese

**Affiliations:** 1 Department of Mechanical Engineering, Aeronautics Advanced Manufacturing Center (CFAA), Faculty of Engineering of Bilbao, Alameda de Urquijo s/n, 48013 Bilbao, Spain; norberto.lzlacalle@ehu.eus; 2 Centro de Investigación y Desarrollo en Mecánica, Instituto Nacional de Tecnología Industrial INTI, Avenida General Paz 5445, 1650 Miguelete, Provincia de Buenos Aires, Argentina; vmart@inti.gob.ar (V.M.); mkrahmer@inti.gob.ar (D.M.K.); 3 Centro de Investigación y Desarrollo en Construcciones, Instituto Nacional de Tecnología Industrial, Avenida General Paz 5445, 1650 Miguelete, Provincia de Buenos Aires, Argentina; alemir@inti.gob.ar; 4 All Import SA, Paraná 4532, 1605 Munro, Provincia de Buenos Aires, Argentina; gustavo@e-bremen.com

**Keywords:** dry-cutting, concrete, segmented diamond blade, topography, diameter variation, weight loss

## Abstract

The objective of the present study is to analyze and compare the cutting performance of segmented diamond blades when dry-cutting concrete. A cutting criteria is proposed to characterize the wear of the blades by measuring the variation of the external diameter and the weight loss of the blade. The results exhibit the cutting blade SB-A, which has twice the density of diamonds and large contact area, exhibits less wear even though the material removal rate is higher compared with the other two cutting blades. Additionally, the surface topography of the different blades is evaluated to examine the impact of wear depending on the surface profile and the distribution of the diamonds in the blade’s matrix. Large number of diamonds pull-out are found in blades type SB-C, which additionally shows the worst wear resistant capability. As a conclusion, the cutting efficiency of the blade is found to be related to the density of embedded diamonds and the type of the surface profile of the cutting blade after reaching the stop criteria.

## 1. Introduction

Diamond blades were designed to cut hard or abrasive materials such as concrete, marble and ceramics. During the last 15 years, blades with diamond segments (diamond particles embedded in a cementing matrix) have allowed substantially increasing the effectiveness of these cutting operations [1]. Generally, researchers have focused on two topics when studying the performance of diamond blades: the first topic deals with the lifespan and tool wear, while the second topic studies the effectiveness in term of energy consumption under different operational configurations, such as cutting forces, vibrations and influence of lubricants. Regarding to the first topic, González et al. [2] described analytical model to predict wear of different metal alloys during a cutting process. They took into account the relative speed of the abrasive material, the applied force, the type of material and the size of the abrasive grains to describe the abrasive mechanism. Consequently, wear coefficients are proposed that behave linearly with the size of the abrasive grain for each metal alloy. Sánchez et al. [3] described a novel dressing method to recovery large-size grinding wheels. In this sense, a specific electrical mechanism to heal the conductive-bond with several electric variables such as electrode size, wheel speed and several current configurations were proposed. They found that the current configurations mainly affect the material removal rate, whereas the other parameters are linked to the process capability. Grinding experiments showed that this novel method can reduce up to 50% of the cutting forces and, ultimately, higher depth of cut can be assessed. Furthermore, Lopez de Lacalle et al. [4] focused on study how coating improves the specific cutting force on high speed steel (HSS) and cemented carbide flying tools while cutting wood. As a result, an increase of the lifespan of 116% and more than 30 km of cutting length was achieved with the HSS tool coated with TiCN or AlCrSiN. Recently, Guerra Rosa and coworkers [5,6,7] investigated the relationship between wear and the mechanical parameters of different metallic binders utilized to manufacture cutting tools. In particular, they studied the wear behavior of powder metallurgy matrices used in diamond tools and the possible relationship with their mechanical properties. The wear behavior of these matrices was evaluated during the processing of some common polycarbonate and soda-lime glass plates, and also in much harder materials, e.g., granite tiles. The results showed a linear relationship of the wear with the normal force, whereas the wear evolution of these matrices processing granite was about 100 times higher than processing glass; and in glass it was approximately 10 times higher than processing polycarbonate. Even though the mentioned literature presented the wear evolution and the behavior of the lifespan of different cutting tools, there is no standardized test in the industry to evaluate the performance of the cutting discs under common operational configurations.

Much research has been published about the cutting effectiveness using different operational configuration. For example, vibration issues when high-speed cutting stone with large diamond blades are commonly found [8]. The effects of the outer and inner diameters of the blade can be determined in the frequency spectrum, whereas the size of blades is linearly related to the spindle speed, which induces resonance and high vibrations. This issue can be reduced with a proper selection of the operating conditions and by increasing the stiffness and/or the internal strength of the blade. Additionally, Ucun and coworkers [9] investigated the diamond density and the type of matrix material when cutting granite stone. During the experiment the cutting forces and the specific cutting energy were analyzed to enhance the cutting performance. They concluded that using a high ratio of W-Co as a matrix compound enhance the cutting efficiency by reducing the tool wear, the cutting forces and the specific cutting energy. Slight improvement of wear was found when high diamond density was assessed in the segment area. Few years later, they [10] studied the influence of four lubricants when cutting stones with diamond blades to compare the results with respect to the same cutting process but employing water as a lubricant. They found that water mixed with boron oil achieved lower power consumption and higher wear resistance of the diamond blade. Furthermore, Oliveira et al. [11] analyzed several metallic matrices with embedded diamonds during sawing process. In particular, they analyzed the cutting forces and the tool wear when different iron-based powders were used to prevent diamond pull-outs. Consequently, lower tool wear was denoted with these type of powders compared with a standard tool, although higher cutting forces were found due to a higher number of diamonds at the contact surface. Regarding the cutting forces, the influence of the axial cutting force on the tool wear and lifespan of the blade when cutting marble was addressed [12]. The increasing the feed rate and depth of cut are associated with an axial deviation, which can promote circular cracks in the flange region. Moreover, the cutting capabilities of different diamond blades and metallic matrices were investigated by using several working parameters when cutting two types of stones (limestone and marble) [13,14]. During the cutting tests, vertical and horizontal forces, electric energy consumption and vibrations were analyzed with the aim to evaluate and, ultimately, the efficiency of the cutting tool was characterized according to the mechanical properties of the different binders, the size of diamond grit and type of the bounding matrix used to embed the diamonds. Furthermore, Konstanty and Tyrala [15] described the wear mechanism of cutting tool with diamonds embedded under real industrial conditions. This work revealed that the wear rate was major affected due to the diamond density and the iron-base matrix compound exhibit the best cutting performance results against wear. Finally, they described the interaction between the diamonds and the surface to cut, denoting the importance of a matrix to retain the diamond during the cutting process to avoid pull-outs.

In addition, Ucun et al. [16] examined the energy consumption variation according to several cutting parameters when cutting natural stones with diamond blades. During the cutting process, down cutting was performed as the cutting operation, granite (Blue Pearl) was used as the natural stone and water was preferred as the cutting lubricant. Three different cutting parameters, depth of cut, circular velocity and cutting speed, were chosen for the cutting experiments. Specific cutting energy values were obtained through an analytic method using the data recorded from the energy analyzer and the dynamometer. According to these results, the power consumption increased as the cutting parameters increased, but the specific cutting energy decreased. Therefore, the depth of cut was the most sensitive cutting parameter for the rise of power consumption. Accordingly, a new computer algorithm to maximize tool productivity of a sawing process is addressed, taking into account the stone characteristics and the quality required for the end product [17]. This algorithm essentially depends on three variables, namely, the cutting depth, the feed rate and the rotational velocity, as well as how these variables are related with the forces acting on the tool. Oliveira et al. [18] examined the viability of adding different additives to the Fe-Cu matrix to make a binder material with the adequate wear properties needed for manufacturing diamond impregnated tools for cutting stone. After the hot-pressing cycles, the main mechanical properties of the sintered bodies were evaluated. Cutting tests under real conditions with Porriño granite were carried out to compare the performance of the tools. The results from the cutting trials revealed that the tools show quite similar behavior during the cutting operations, thus indicating that replacement of Nb with Co is a promising challenge to be followed in the near future. Recently, Krolczyk and coworkers [19,20] investigated in detail measurement protocols of effective areas of different cutting tools and propose which are the advantages and limitations of the optical measurement devices depending on the type of abrasive tool. Additionally, they stated the differences of dry cutting and cutting with lubricant and the onset wear effects in the effective cutting area such as adherence and cracking. As a result, they found that the surface profile after the cutting process revealed higher tribological irregularities when machining without coolant, subsequently higher plastic flow at the cutting zone. Finally, Hu et al. [21] studied the cutting performance of three kinds of diamond blades with different structure parameters. Sawing force and vibration were measured by cutting several concretes with different strengths with different cutting parameters. Later, the characterization of the sawing forces and vibrations helped to find the optimal structure of diamond blade to properly operate with the following parameters: segment width, sawing velocity and the type of the material to cut. Following this research topic, the aim of this work is to evaluate and compare the cutting performance of segmented diamond blades when dry-cutting concrete, where the stop cutting criterion is fixed based on the total surface of concrete to cut. Likewise, the tool wear is measured by the variation of the external diameter and the weight loss of the blade. The final topography of the blades is evaluated to determine the impact of wear at the surface and, ultimately, the integration of the diamonds in the matrix. In this sense, it is possible to quantify when a blade possesses a superior cutting capability, in terms of the common machining operation without risking the lifespan of the cutting machine.

## 2. Methodology

**Figure 1:**
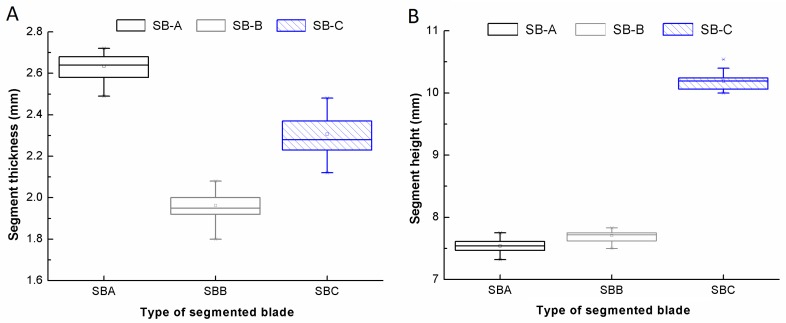
Boxplot of the: thickness (**A**); and height (**B**) of the cutting segments for each type of diamond blades.

A total of 12 universal Segmented Blades (SB-A, SB-B and SB-C, four samples per brand) with embedded diamonds were investigated. The specifications of the blades are: 115 mm of diameter, 13,200 rpm of maximum spindle speed, 80 m/s of maximum cutting velocity and all the blades fulfilled the EN 13236 standards. The three segmented blades SB-A, SB-B and SB-C dispose 9 segments for the first two models and 8 segments for SB-C model. Figure 1 shows two box plots with the geometrical measurements of thickness and height of each of the cutting segments for each type of diamond blade. Lines within the box plots represent the median; squares represent the average; whiskers represent the tenth and ninetieth percentiles; and crosses below and above the box plots represent maximum and minimum sided values.

A rectangular mortar matrix was designed and developed to fabricate the concrete blocks by following the standard IRAM 1534:2004. A total of 65 prismatic blocks of 290 × 260 × 40 mm^3^ were developed by mixing concrete with medium size stones. Particularly, the composition of the concrete was 170 kg/m^3^ of water, 350 kg/m^3^ of concrete Portland CPC-40, 800 kg/m^3^ of siliceous sand, 191 kg/m^3^ of granitic sand (dimension up to 6 mm), 875 kg/m^3^ of medium size stones (dimension of 6 to 12 mm), 1% and 0.55% of mass of cement of two superplasticizer additives Mira-57 and Daracem-18, respectively. The procedure for preparing the specimens was the following. Firstly, the sand and gravels were combined in the concrete mixer during 30 s. Then, the Portland cement was added and mixed during 30 s. Subsequently, the half part of the water and the additives were added and combined during 90 s. Finally, the rest of water and additives were introduced and mixed in the mixer during 180 s. Following this procedure, a homogeneous blend was obtained. Later, the mechanical characterization of the concrete was performed with compression tests of 8 cylindrical specimens of Φ 150 × 300 mm^2^ with 28 days of curation following the standard IRAM 1546:1992. The average of the ultimate tensile strength of the concrete blocks was found to be 39.1 ± 1.2 MPa, which is the crucial parameter to evaluate the cutting performance of the diamond blades.

**Figure 2:**
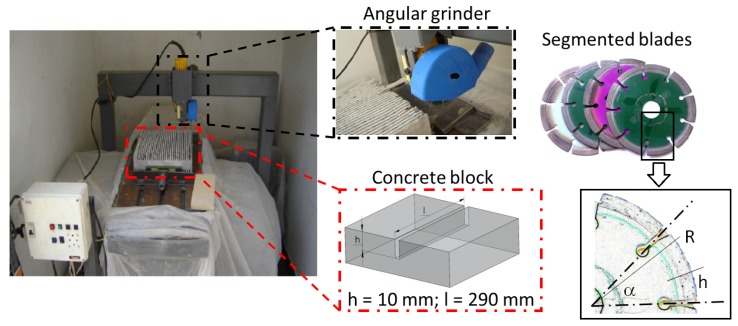
Cutting process of dry-concrete by using different segmented diamond blades in an in-house developed automatic machine.

All the linear cuts were made on the two opposite faces of the concrete blocks with greater longitudinal area. A total of 28 linear cuts with a lateral displacement of 9 mm were performed in each face. A cutting area of 650,000 mm^2^ (0.65 m^2^) was established as the total length or the stop criteria to measure the wear of the blades; similar values were used by Oliveira et al. [18]. This area is equivalent to make 56 cuts of 10 mm deep and 290 mm long in 4 different concrete blocks. An in-house adapted bench was utilized to accurately perform all these linear cuts. This bench can move in two axes of the horizontal plane due to a pair of servomotors which are regulated with a programmable control. A manual angular saw (Makita, model: 9557HPG, Anjō, Japan) of 840 W and 11,000 rpm was attached in a structural bridge and, then, allocated above the bench. The depth of cut was manually set with a height adjustment module. To select the proper operational cutting parameters, calibration tests were carried out to define the maximum feed rate for a depth of cut of 10 mm and without exceeding a consumption of 7 A. As a result, the feed rate selected to perform the cutting experiments was 320 mm/min. Figure 2 exhibits a schematic diagram with pictures of the in-house cutting machine of concrete blocks with segmented diamond blades.

The thickness and height of the segment area were assessed using a digital caliber (Tesa Technology, model: 00530084, Renens, Switzerland). The same caliber was employed to measure the diameters of blades before and after the cutting experiment. An electronic scale (Moretti, model: OAC 2.4, South Deerfield, MA, USA) with an accuracy of 0.2 g was utilized to record the weights of the as-received blade and after reaching the stop criteria. Additionally, optical microscope (Carl Zeiss, model: Axiotech 100HD-3D, Oberkochen, Germany) and electron microscopy (Philips, model: SEM 505, Amsterdam, The Netherlands) were employed to capture images of the cutting characteristics and the diamond size of each blade. Finally, a 3D profilometer (Leica, model: DCM3D, UPV, Bilbao, Spain) with a HCX PL Fluotar objectives of 10× magnification was used to measure the surface and profile topographies of the segment area. The resolution was 140 nm in-plane and 100 nm in vertical direction by using confocal technique. The experiments were accomplished at the INTI-Mechanics Center in Argentina.

## 3. Results

### 3.1. Cutting Tool Characterization

Firstly, the density of diamonds in each segmented blade is measured to determine if significant differences are presented between the studied blades. To do that, the diamonds expose on both sides of the segment area are counted. Table 1 shows the number of diamonds measured on both side of the segment, the average number of diamonds per blade and the error dispersion.

Regarding the blades differences, it is desired to estimate the density of diamonds in each blade. To do that, the effective area of each segmented blade type is calculated as follow:

(1) $$A_{s}=\frac{\alpha }{360}\cdot \left[\pi \cdot \left(R^{2}-{\left(R-h\right)}^{2}\right)\right]$$

where $A_{s}$ is the segment area, *R* is the radio of the blade, *h* is the segment height measured in Figure 1, and α is the effective angle of the segments area which depend on the type of blade used. Later, the density of the diamond per segment area is determined by multiplying the effective segment area per the average diamond quantity in a single segment. Table 2 shows the estimated density of diamonds per effective segment area in each blade.

**Figure 3:**
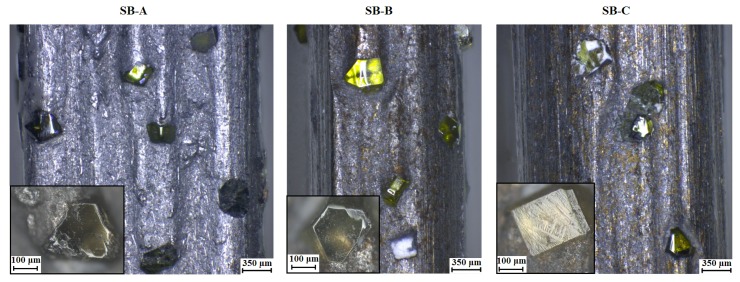
Grain size, thickness and matrix–diamond distribution for each type of blade.

The density values show that the SB-A blade dispose twice the number of diamonds in a segmented area compared to the SB-C blade. Many diamonds embedded in the blade’s matrix increase the cutting efficiency and let us increase the feed rate to cut rough grain structure such as concrete [21]. Therefore, higher diamond density in the segment area is expected to extend the lifespan of blade due to decrease the average cutting force of each diamond, despite that increasing the number of diamond will increase the cost of blade. To characterize the profile of the matrix–diamonds distribution of each blade, a segment area of each blade was examined by electron microscopy. Figure 3 exhibits the matrix–diamond distribution for each type of blade.

Five measurements of the diamond size were taken to determine that the diamond dimension are equivalent among the three blades. In this sense, the diamond size are 0.26 ± 0.11 mm, 0.36 ± 0.08 mm, and 0.27 ± 0.08 mm for the blade type SB-A, SB-B and SB-C, respectively. Figure 3 shows that many diamonds are found on the surface of SB-A compared to the other two types. Therefore, more diamonds will be in contact with the surface of the concrete to cut; thus, a higher cutting efficiency is expected during the process.

Finally, the material removal rate is computed for each diamond blade to compare the tool wear with comparable cutting configurations. Accordingly, the material removal rate is a function of the cutting conditions and of the operation performed. For a sawing operation, the material removal rate (*MRR*) can be determined as follow:

(2) $$MRR=f\cdot A_{c}=f\cdot \left(\frac{\pi \cdot R^{2}}{2}+w\cdot \left(d-r\right)\right)$$

where *f* is the feed rate (mm/min), $A_{c}$ is the effective cross sectional area, *R* is the radio of the blade, *w* is the average segment width measured in Figure 1, and *d* is the depth of cut set during the cutting trials. As an approximation, the area of the blade’s edge was considered as a semicircle of diameter equal to the width. Subsequently, the values of the material removal rate are 22.0 mm^3^/min, 19.3 mm^3^/min and 19.8 mm^3^/min for the blade type SB-A, SB-B and SB-C, respectively. Therefore, SB-A is cutting more concrete in the same time and, consequently, a higher tool wear is expected compared to SB-B and SB-C.

### 3.2. Tool Wear Measurement

As mentioned above, 0.65 m^2^ of cutting area was assessed as the total length/stop criteria to measure the wear of each blade. Two different ways of measuring the tool wear are commonly used: diameter difference and weight loss of the blade. The aim here is to determine if both methods for measuring the tool wear are comparable. Firstly, the tool wear is estimated by measuring the difference of the initial diameter and diameter of blade once the stop criteria is assessed, as listed in Table 3.

On the other hand, the tool wear is also estimated by measuring the difference of the initial weight and weight of blade once the stop criteria is reached, as listed in Table 4.

**Figure 4:**
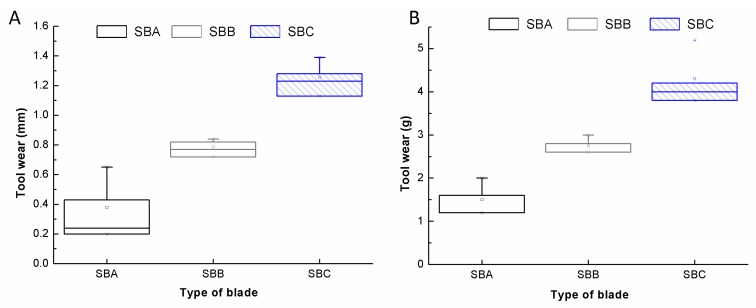
Boxplots of the tool wear in the three studied diamond blades when reaching the stop criteria in terms of: diameter variation (**A**); and weight loss (**B**).

Boxplots are assessed to address the dispersion of the diameter variation and weight loss and, subsequently, to statistically compare the tool wear differences between the three blades. Figure 4 shows the tool wear in terms of diameter variation and weight loss when reaching the stop criteria of the cutting length.

**Figure 5:**
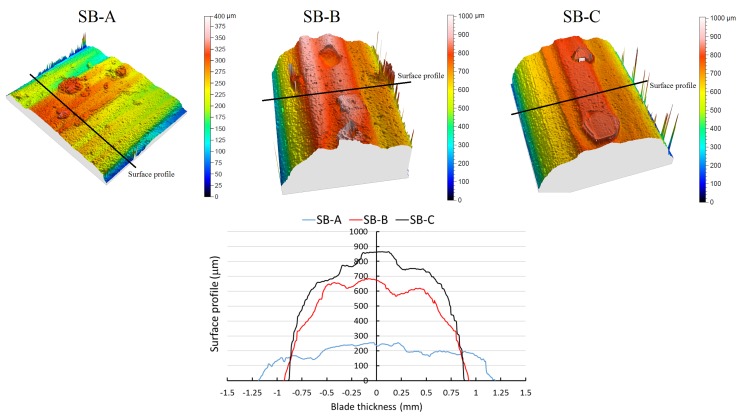
Surface topography of the edge of the segment area for each type of blade.

From the boxplot diagrams of diameter variation and weight loss, it is clear that wear differences are found in each blade. Lowest values of wear are found in SB-A compared with SB-B and SB-C. On the contrary, wear values of 1.3 mm of diameter difference and about 4 g of weight loss is recorded in the SB-C type after completing the cutting criteria. SB-A, SB-B and SB-C were staggered in an increasing wear sequence. Consequently, the blade type SB-C shows the worse behavior against wear, exhibiting a diameter change of 1.26 mm and a weight loss of 4.30 g on average. On the other hand, SB-A exhibits the best performance with a diameter variation of 0.38 mm and a weight loss of 1.50 g on average. Consequently, the results show that tool wear can be measured either by recording the weight loss difference or by measuring the diameter variation, as both methods have been found to be equivalent. To study in detail the different tool wear findings, the surface topography was evaluated to analyze the surface profile of each blade. Figure 5 exhibits the surface of the edge of the segment area for the three blades, SB-A, SB-B, and SB-C, after reaching the stop criteria. The peaks found in the contour of the surface topography is noise due to the side-effects from the optical acquisition procedure.

The results show that the topography of a surface is different for each case, as expected from the wear resistant values found in Figure 4. In particular, the SB-A presents a higher flatness surface and, consequently, more diamonds in the surface compared to the other two blades. This type of surface will bring a higher contact area during the cutting process, which means higher cutting forces due to friction. A higher cutting efficiency is found due to high number of diamonds in contact with the concrete during the dry-cutting process. A similar conclusion was stated by Turchetta [22], where he described that cutting forces and the specific cutting energy increase depending on the shape of the interface between cutting tool and workpiece, as a higher material removal rate is achieved. SB-B and SB-C show similar surface profiles within the range of 700–800 µm of maximum height and about 2 mm of thickness. Both cases exhibit that the diamonds are allocated in the center of the blades thickness and the agglutinant is wearing on both sides of the blade, leaving a prominent semicircle in the cross sectional area of the segment area. During the sawing process, it is crucial the compound matrix of the segments area keep the diamond grains for as long as possible during the cutting process. The blade type SB-C was analyzed using an electronic microscope after finishing the cutting criteria to determine if any evidence can explain the wear differences, in terms of diameter and weight, between SB-B and SB-C. Many holes due to diamond grain pull-out were observed in the SB-C. This loss of diamond grains in the agglutinant clearly affects the cutting capacity of the blade and enhances the tool wear. No diamond detachment occurred in cutting blades SB-A and SB-B. It is possible to minimize the diamond grit pull-outs by using active elements in bounding matrix (Bailey and Collin [23]), such as Cr and Co, which have been found to enhance the diamond holding effectiveness and improve wear performance of the diamond disc [18].

In future work, the cutting forces will be recorded when cutting dry-cutting concrete with the aim of estimating the power consumption and material removal rate for each type of blade. It is expected that, assuming the same depth of cut, the surface topography will modify the cutting forces and, consequently, the power consumption of the saw. Additionally, the wear evolution will be analyzed by measuring the diameter variation and weight loss for different cutting length values until reaching the failure of the cutting tool. Accordingly, both the wear evolution and the lifespan of the segmented diamond blades can be characterized. Finally, several bounding matrices and surfaces coatings, such as TiN and AlTiN, will be characterized to address the adhesion of diamonds to improve the wear resistance of these cutting tools.

## 4. Conclusions

The present work describes a procedure to evaluate the wear performance of different segmented diamond blades. The following findings can be withdrawn:

- It is necessary to adapt a cutting machine to test several cutting conditions with a previously defined stop criteria and, ultimately, measure the tool wear according to the tool diameter variation and the weight loss of the blade.
- The density of the diamonds in a segmented area is experimentally estimated by dividing the number of diamonds found in the segment surface per the effective segment areas of each type of blade. The wear experiments show that the diamond density exhibits a strong impact on the cutting efficiency of the blade.
- The surface profile seems to influence of the cutting capability, where a higher surface flatness enhances the cutting efficiency, due to higher contact area of the surface to cut with the diamonds grains embedded in the metallic matrix.
- Larger cutting areas and higher cutting feed rate are necessary to evaluate the wear performance of the blades as well as the wear capability of the blades before the failure.
- The limitation of the presented study deals with the bounding matrix composition of each blade, which is unknown. Likely, the diamond pull-out found in SB-C blades is due to not using effective active elements in the bounding matrix. Unfortunately, this information is neither provided by the supplier nor listed in the technical data sheet of the product.

## Figures and Tables

**Table 1:** Quantity of diamonds per segment of each type of blade.

Parameter/Blade Type	SB-A	SB-B	SB-C
Sample 1	57	36	42
	54	32	52
	63	46	50
	59	40	48
Sample 2	55	49	56
	61	35	51
	59	56	35
	54	24	35
Sample 3	49	45	42
	50	32	42
	75	38	40
	55	50	37
Sample 4	74	46	48
	48	50	47
	40	31	52
	53	36	70
Average	56.63	40.38	46.69
Dispersion	8.93	8.79	8.93

**Table 2:** Estimated density of diamonds per effective segment area of each blade.

Parameter/Blade Type	SB-A	SB-B	SB-C
Av. segment height (mm)	7.54	7.71	10.19
Number of segments	9	9	8
Effective angle of area (°)	33.50	37.50	53.30
Effective segment area (mm^2^)	237.69	265.43	377.33
Av. diamond quantity per segment (n° grains)	56.62	40.37	46.68
Diamond density per segment (n° grains/mm^2^)	0.24	0.15	0.12

**Table 3:** Tool wear analyses by measuring the diameter difference.

Blade Type/Parameter	Initial Diameter	Final Diameter	Difference	Average	Dispersion *
	(mm)	(mm)	(mm)	(mm)	(mm)
SB-A	118.45	118.25	0.20	0.38	0.21
	118.49	118.25	0.24		
	118.25	117.60	0.65		
	118.61	118.18	0.43		
SB-B	115.07	114.35	0.72	0.79	0.05
	115.29	114.45	0.84		
	115.25	114.48	0.77		
	115.02	114.20	0.82		
SB-C	115.39	114.00	1.39	1.26	0.11
	115.52	114.24	1.28		
	115.32	114.19	1.13		
	115.49	114.26	1.23		

* The error dispersion is estimated with the standard deviation.

**Table 4:** Tool wear analyses by measuring the weight loss difference.

Blade Type/Parameter	Initial Weight	Final Weight	Difference	Average	Dispersion *
	(g)	(g)	(g)	(g)	(g)
SB-A	158.2	157.0	1.2	1.50	0.38
	158.4	157.2	1.2		
	160.2	158.2	2.0		
	160.8	159.2	1.6		
SB-B	96.1	94.2	2.6	2.75	0.19
	96.8	93.8	3.0		
	97.6	94.8	2.8		
	95.2	92.6	2.6		
SB-C	106.4	101.2	5.2	4.30	0.62
	103.6	99.8	3.8		
	105.8	101.8	4.0		
	104.2	100.0	4.2		

* The error dispersion is estimated with the standard deviation.
